# Female (Under) Representation in Exercise Thermoregulation Research

**DOI:** 10.1186/s40798-021-00334-6

**Published:** 2021-06-22

**Authors:** Kate P. Hutchins, David N. Borg, Aaron J. E. Bach, Joshua J. Bon, Geoffrey M. Minett, Ian B. Stewart

**Affiliations:** 1 Institute of Health and Biomedical Innovation, School of Exercise and Nutrition Sciences, Queensland University of Technology, Brisbane, Australia; 2 The Hopkins Centre, Menzies Health Institute Queensland, Griffith University, Brisbane, Australia; 3 The National Climate Change Adaption Research Facility, Griffith University, Gold Coast, Australia; 4 School of Mathematical Sciences, Queensland University of Technology, Brisbane, Australia; 5 Australian Centre of Excellence for Mathematical and Statistical Frontiers, Brisbane, Australia

**Keywords:** Exertional heat stress, Menstrual cycle, Performance, Sex differences, Temperature

## Abstract

**Background:**

Despite an increasing rate of women participating in professional sports, emergency services, and military settings where they are exposed to exertional heat stress, our understanding of female thermoregulation and the detrimental effects of heat on women’s performance, especially regarding the menstrual cycle, is limited. This review aimed to quantify the representation of women in exercise thermoregulation research between 2010 and 2019 and the frequency that these articles reported details pertaining to female participants’ menstrual cycle to determine the volume of novel research that is directly relevant to this growing population.

**Methods:**

Original exercise thermoregulatory studies published in three major sports medicine databases (PubMed, MEDLINE, and SPORTDiscus) between 2010 and 2019 were surveyed. Articles were screened to determine the number of female and male participants in the study and whether studies involving women reported menstrual orientation or phase. Research involving healthy adult participants and an exercise protocol with a thermoregulatory outcome measure were included in the review.

**Results:**

A total of 1407 articles were included in the review, involving 28,030 participants. The annual representation of women ranged from a mean of 11.6% [95% credible interval (CI); 9.2, 14.3] to 17.8% [95% CI; 15.2, 20.6] across the 10 years, indicating studies predominantly included men. Nonetheless, there was a small statistical increase in the overall proportion of women, with a mean overall proportion change of 0.7% [95% CI; 0.2, 1.2] per year. The increase appeared to be driven by a reduction in the number of studies including only men, rather than studies including more women alongside men, or increased women-only studies. Less than one third of articles involving women reported the menstrual orientation of participants and less than one quarter reported both menstrual orientation and phase.

**Summary/Conclusion:**

This study shows that women were proportionally underrepresented in exercise thermoregulation research during the past decade and the majority of studies did not report menstrual cycle details of female participants. Researchers should consider including women in future work where their inclusion could contribute meaningful data that enhance the evidence-based and ultimately improves our comprehension of women’s thermal physiology.

**Supplementary Information:**

The online version contains supplementary material available at 10.1186/s40798-021-00334-6.

## Key Points

- Women are significantly underrepresented in the exercise thermoregulation literature over the past decade, accounting for only 30% of the total participants in 2019.
- The study of women’s exercise thermoregulation increased between 2010 and 2019, and this proportional change was explained by less men-only studies rather than increased involvement of women.
- Instead of independent study, under-representative female samples are included in mixed-sex groups, with the median sample size of women and men subgroups being six and ten, respectively.
- Despite limited and conflicting data on the effect of the menstrual cycle on thermoregulatory functions, less than 30% of articles reported women’s menstrual orientations and only 22% reported both menstrual orientation and phase.

## Background

Males and females are defined by their innate anatomical and physiological characteristics [1, 2]. Each sex is often studied independently, especially in exercise science and sports medicine, to avoid sex-related confounding effects [3, 4]. For example, disparities between the sexes have been shown with muscle-tendon adaptations [5], injury susceptibility [6, 7], and decision making under pressure [8]. Interestingly, both similarities and differences between the sexes are evidenced in the exercise thermoregulation literature.

Men and women respond to heat stress much the same when the rate of metabolic heat production is appropriately fixed [9, 10]. Individual variability in temperature regulation during tasks without a fixed internal heat load is mainly due to morphological and fitness-related characteristics altering internal heat production and the heat loss required to attain heat balance [9, 11]. Sex differences are noted in the size and density of sweat glands [12, 13] and sweat output at elevated requirements for heat loss [9, 14]. In women, the thermoregulatory system is also in a continuous state of change across the menstrual cycle. As the concentrations of reproductive hormones shift, so too does core temperature, along with modifications to the onset thresholds and sensitivity of autonomic heat loss responses [15–17]. Multiple recent works have failed to reveal any large effects of the menstrual cycle on heat dissipation during exercise tasks [18–20]. However, given limited data availability and the poor validity of some previous studies [18], the certainty of this conclusion is not without reservation. Thus, it remains undetermined if sex-based differences relating to the sudomotor function and the menstrual cycle make women more susceptible to performance deficits in the heat [21–23]. With more women entering physical occupations [24, 25], a drive for greater representation in emergency services [26, 27], increased professionalism of women in elite sport [28, 29], and extreme heat becoming more frequent [30], there is an impending need for further knowledge.

Anecdotally, exercise thermoregulation studies involving women appear to be growing. However, this has not been empirically confirmed. The sex bias and general omission of women reported in sports science literature [31] are also likely to be true in the area of exercise thermoregulation. High-quality thermoregulation studies involve complex study designs and will often require repeated data collection periods for participants. The additional time and resources required to appropriately control for any contraceptive, hormonal, or menstrual cycle influences in this field have likely contributed to the absence of women in previous work [32]. This study aimed to systematically review the representation of women in exercise thermoregulation literature between 2010 and 2019 to provide perspective on modern research practices. It was hypothesized that the proportion of women included in studies has not changed over this period. A concomitant aim was to determine how frequently these articles reported details pertaining to female participants’ menstrual cycle or phase.

## Methods

### Search Strategy

The search was conducted according to the conventional systematic review processes to select appropriate literature and ensure the search reproducibility [33]. A list of keywords relevant to the research theme was created with the assistance of MeSH browsers, trialed, and refined until the most effective search statement was achieved. Three major sports medicine databases—PubMed, MEDLINE, and SPORTDiscus—were searched using the key terms, Boolean operators, and limits. The final list of search terms and complete search statement is provided in Supplementary 1.

### Inclusion Criteria

Included studies (a) were published between January 2010 and December 2019, (b) had an accessible English abstract, (c) included original data (d) from healthy (i.e., free of acute or chronic disease) adult humans (≥18 years), (e) had a design that included any form of exercise or movement in the protocol, and (f) described a thermoregulatory outcome measure (Supplementary 1). Participants from studies using healthy control groups were included in the results.

### Screening

**Fig. 1:**
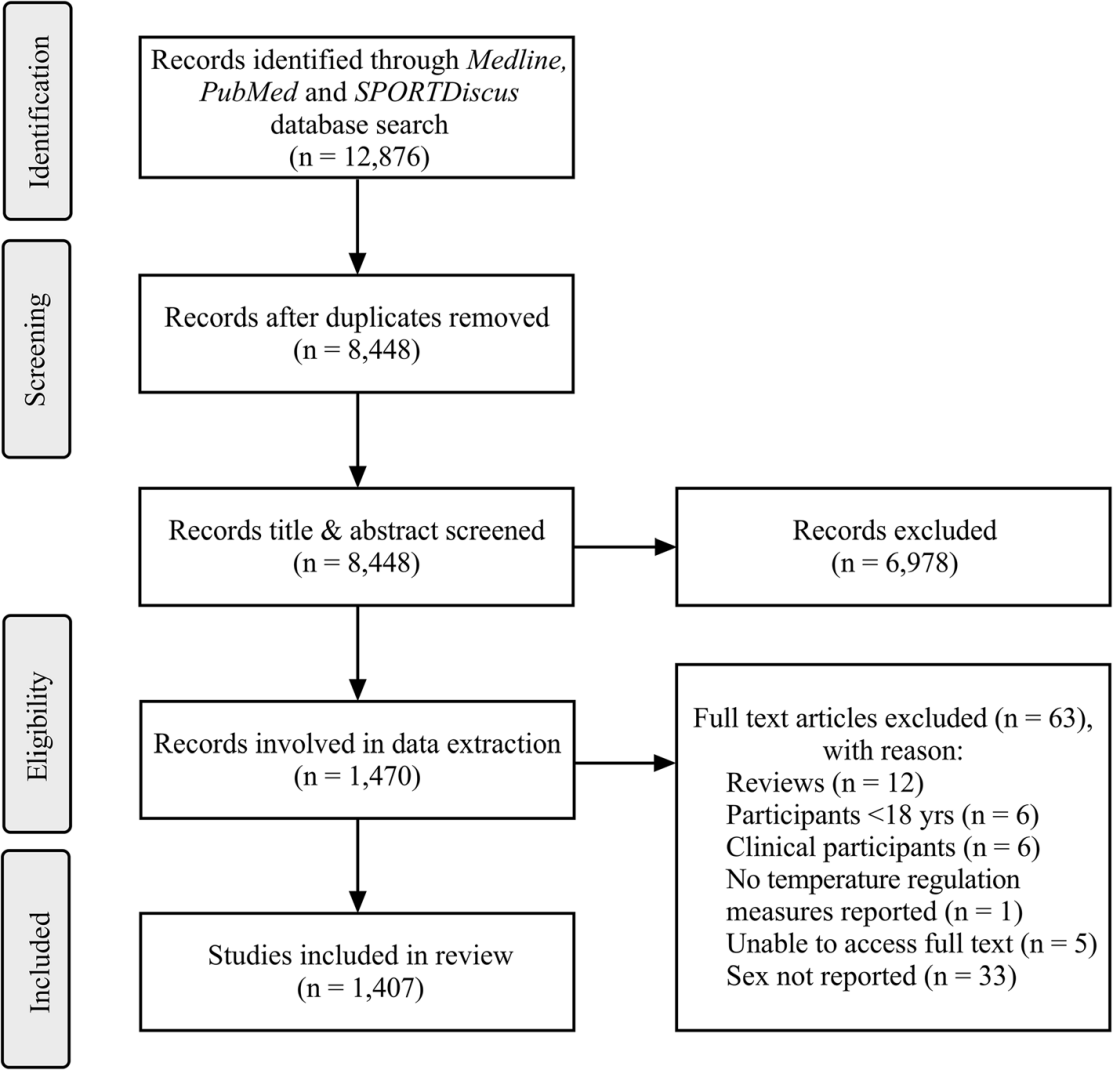
PRISMA flow diagram of record search and data extraction

A total of 12,876 results were retrieved from the database searches on December 10, 2019—5818 from PubMed, 5917 from MEDLINE, and 1141 from SPORTDiscus (Fig. 1). Results were imported into Covidence (Covidence Systematics Review Software 2019, Veritas Health Innovation). Duplicates were removed, and titles and abstracts were screened. Two authors independently screened all articles against the inclusion criteria. Conflicts were resolved by a third author. A PRISMA flow diagram summarising the search process is provided in Fig. 1 [33].

### Data Extraction

Eligible studies were exported to Excel (16.0, Microsoft Corp., Washington, USA), where the number of women and men in each study was extracted from the full text. Corresponding authors were contacted to clarify participant numbers and sex if such details were not explicitly stated in full text. The authors acknowledge that an individual's sex can change from birth, as in the case of transgender people. As such, the authors recorded if participants were specified to be transgender women or men and included them in the count of women and men respectively in the reviewed studies.

Secondary data extraction was completed on studies that involved women. From these studies, the following details were extracted whether menstrual orientation (i.e., natural menstruating, hormonal contraceptive user, pregnant, postmenopausal, oligomenorrheic, secondary amenorrheic cycles) was reported, the number of participants reported in these groups, and whether menstrual cycle phase was reported. The extraction process was completed by two authors. A list of the included studies can be accessed at https://github.com/katehutchins/female-thermoreg-review.

### Data Analysis

Descriptive statistics are reported as count and percent. The median (interquartile range; IQR) sample size of studies with only women, both men and women, and only men was calculated. The proportion of women included in studies was calculated using the equation: *n*/*N*, where *n* is the number of women and *N* is the total number of participants in the study. A proportion of 0 would indicate no women were included in a study, with 1 indicating only women were included. Zero- and one-inflated beta (ZOIB) regression was used to investigate whether there was no change in the proportion of women included in studies between 2010 and 2019 [34]. A beta regression can model values between 0 and 1 (exclusive) and is often used to model proportional data [34, 35]. ZOIB regression extends beta regression by allowing exact 0s and 1s to be observed, with some unknown probability.

As with generalized linear models (e.g., logistic regression), each parameter governing the ZOIB regression is connected to a linear regression equation (a linear combination of coefficients and covariates) via a link function. In this case, the logit link function was used for all components. The mean of the beta distribution, the probability of observing an exact 0, and the probability of observing an exact 1 were each modeled with a distinct intercept and coefficient for the time covariate (on the linear scale). The shape of the beta distribution was estimated without covariates. For the regression coefficients, a normal (mean 0, precision 0.001) prior distribution was used. Details for the ZOIB model and implementation can be found in Liu and Kong [34], while our R [36] code to replicate the analysis can be found here https://github.com/katehutchins/female-thermoreg-review.

Logistic regression was used to determine whether the reporting of menstrual cycle orientation changed over the decade. For this model, a normal (mean 0, SD 1) prior distribution was used for the regression coefficient (i.e., year). For the ZOIB and logistic regression models, posterior summaries were estimated using Markov chain Monte Carlo (MCMC). Four chains of 250,000 samples were drawn after a burn-in period of 25,000 additional draws per chain. Before summaries from the posterior distribution were calculated, the MCMC chains were thinned by a factor of 10. Regression coefficients (on the logit scale) and proportions are reported as the posterior mean and 95% credible interval (CI).

## Results

**Fig. 2:**
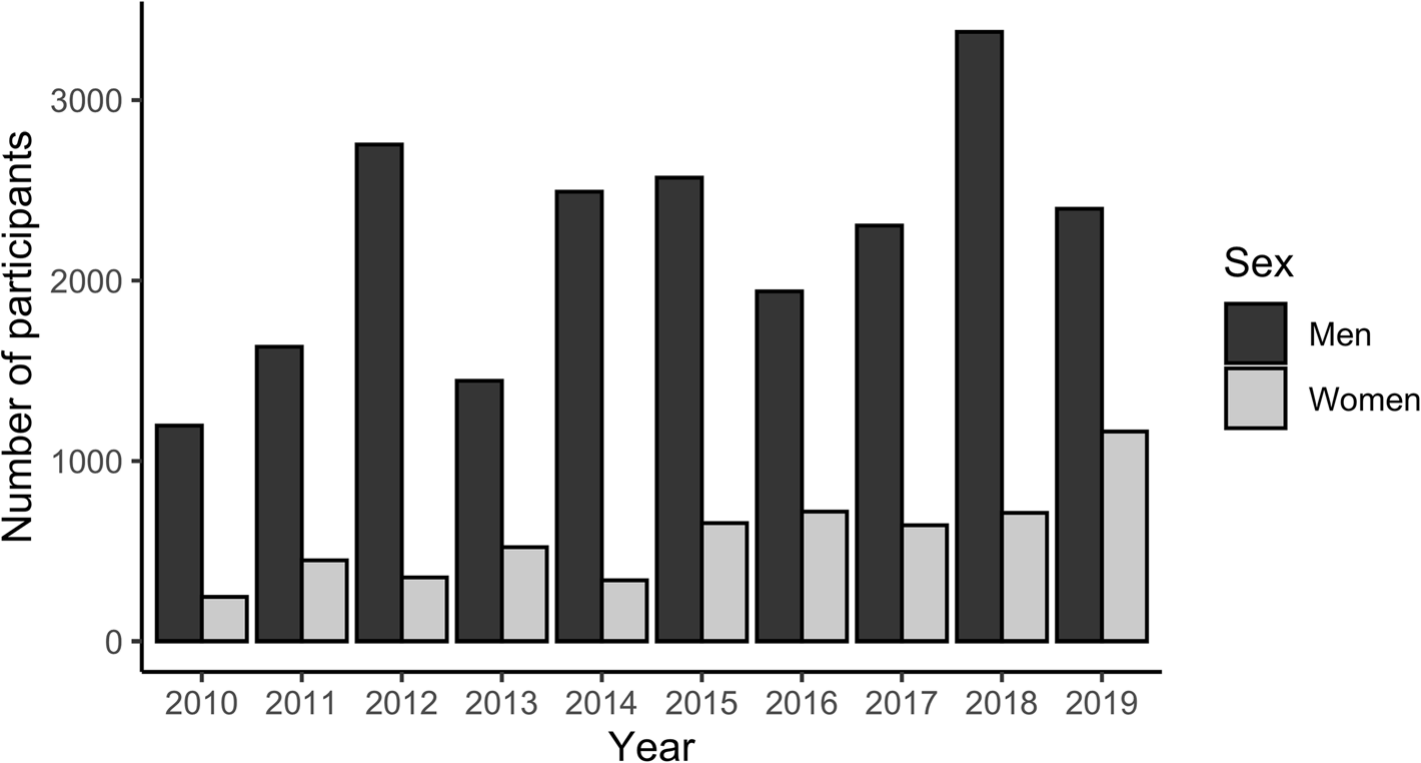
The number of men and women as participants by year

Table 1 summarizes the number of articles each year, Table 2 summarizes the number and breakdown of participants included in these articles, and Fig. 2 depicts the number of men and women by year. The median sample size of studies including only women was 14 (IQR: 10–24). For studies including only men, the median sample size was 10 (IQR: 8–16). In studies including both sexes, the median sample size of men and women subgroups were 10 (IQR: 6–17) and 6 (IQR: 3–10), respectively. One transgender woman was identified and included in the count of women.

**Table 1:** The number of articles total and each group (women only, men only, sex aggregated studies (both)) annually from 2010 to 2019. Percentage relative to the total of each group also presented by year

Year	Annual Articles	Women Only		Men Only		Both	
		Article	%	Article	%	Article	%
2010	95	5	5.3	69	72.6	21	22.1
2011	120	6	5.0	88	73.3	26	21.7
2012	108	7	6.5	81	75.0	20	18.5
2013	117	6	5.1	79	67.5	32	27.4
2014	162	8	5.6	126	77.8	28	17.3
2015	167	6	3.6	120	71.9	41	24.6
2016	134	4	3.0	96	71.6	34	25.4
2017	162	6	3.7	103	63.6	53	32.7
2018	166	11	6.6	111	66.9	44	26.5
2019	176	15	8.5	106	60.2	55	31.3
Decade Average		7.4	5.2	97.9	70.0	35.4	24.7
Standard Deviation		3.3	1.6	18.6	5.4	12.5	5.0

**Table 2:** The number of participants total and each group (women only, men only, sex aggregated studies (both)) annually from 2010 to 2019. Percentage relative to the total of each group also presented by year

Year	Annual Participants	Women Only		Men Only		Both			
		Participants	%	Participants	%	Women	Men	Women %	Men %
2010	1442	71	4.9	945	65.5	174	252	12.1	17.5
2011	2080	173	8.3	1189	57.2	275	443	13.2	21.3
2012	3109	211	6.8	2439	78.4	142	317	4.6	10.2
2013	2009	97	4.8	1010	50.3	469	433	23.3	21.6
2014	2859	112	3.9	2012	70.4	238	497	8.3	17.4
2015	3240	179	5.5	1716	53.0	489	856	15.1	26.4
2016	2667	104	3.9	1213	45.5	623	727	23.4	27.3
2017	2959	69	2.3	1304	44.1	584	1,002	19.7	33.9
2018	4102	304	7.4	2594	63.3	419	785	10.2	19.1
2019	3563	488	13.7	1463	41.1	676	936	19.0	26.3
Decade Average		181	6.2	1589	56.9	409	625	14.9	22.1
Standard Deviation		130	3.2	585	12.3	192	268	6.4	6.6

**Fig. 3:**
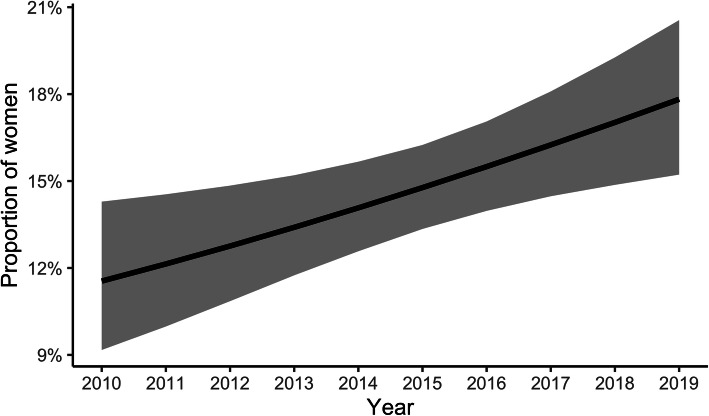
The proportion of women in exercise thermoregulation studies between 2010 and 2019. The dark solid line indicates the mean and the shaded area the 95% credible interval

There was an increase in the overall proportion of women included in exercise thermoregulation research between 2010 and 2019 (Fig. 3), with a mean change in the overall proportion per year of 0.7% [95% CI; 0.2%, 1.2%]. However, in 2019, the estimated proportion was only 17.8% [95% CI; 15.2%, 20.6%] (Fig. 3; Supplementary 2). The increase appeared to be driven by a reduction in the number of studies including no women (*β*_{0}_ [95% CI] = −0.07 [−0.11, −0.03]), rather than studies including a larger subgroup of women (*β*_(0,1)_ [95% CI] = 0.01 [−0.02, 0.04]), or an increase in the number of studies including only women (*β*_{1}_ [95% CI] = −0.01 [−0.10, 0.07]). Supplementary 3 shows the parameter estimates from the zero- and one-inflated beta distribution model.

A total of 422 studies included women. Of these, 72% did not report the menstrual orientation of their female participants. Reporting did not change across the decade (*β* [95% CI] = 0.03 [−0.05, 0.10]). Articles that reported orientation, most frequently involved groups of naturally menstruating women (64%) and oral contraceptive users (26%). Less than 5% of articles identified a group of an intrauterine device or implant users, pregnant or postmenopausal women, or women with secondary amenorrheic or oligomenorrheic cycles. Alongside menstrual orientation, the menstrual phase was controlled in 22% of articles involving women and participants were most often tested in the follicular (48%), placebo pill (21%), or early follicular phase (19%).

## Discussion

This article aimed to offer a perspective on research practices between 2010 and 2019 by determining the proportion of women included in exercise thermoregulation research. As hypothesized, studies predominantly recruited men, with the mean annual representation of women deficient, ranging from 11.6 to 17.8% across the 10 years (Fig. 3; Supplementary 2). Studies generally included women alongside men rather than focusing specifically on women-only cohorts (Table 1). When both sexes were studied together, women represented a lower proportion of the total study sample than men (Table 2). An increase in the proportion of women studied was evident across the decade. Nevertheless, this is explained by a reduction of men-only studies, not greater inclusion of women per se (Supplementary 3). Women were most frequently tested in the follicular phase or placebo stage of the oral contraceptive pill. However, only 22% of studies involving women reported both menstrual orientation and phase. These results indicate that women require greater attention in exercise thermoregulation research.

Historically, the absence of women involved in sport was partly held responsible for the scarcity of sports science research involving female cohorts [37]. Despite increased sex parity in participation rates from grassroots through to the Olympic level [28, 29], women have remained underrepresented in modern sports medicine literature. A review of just three sports medicine journals from 2011 to 2013 found that women accounted for only 39% of the research participants [31]. Similarly, despite growing sport [28] and workplace participation rates [25–27] and the need for a greater evidence-based understanding of women’s heat stress tolerance [18, 38], women continue to be underrepresented in exercise thermoregulation research—both as participants in studies and as the specific subject of scientific inquiry (Table 1, Fig. 2). This review further highlights that women’s representation in this field has not improved across the decade (Fig. 3; Supplementary 2).

Methodological challenges encountered when testing women may explain their large absence from exercise-based thermoregulation research. Studying the interactions between the female reproductive system and thermoregulatory responses is difficult due to the innate complexity of dynamic hormonal shifts [38]. In naturally menstruating women, an elevation in basal core temperature (e.g., 0.3–0.5°C) parallels the luteal phase when progesterone and estrogen peak, whereas a reduced core temperature occurs in the follicular phase when the only estrogen is elevated [39]. The core temperature threshold for autonomic heat loss mechanisms (i.e., peripheral vasodilation and sweating) and response sensitivity is believed to initiate later, at higher core temperatures, and occur at a slower rate during the luteal phase compared to the follicular [40, 41]. This would imply that the biphasic changes could aid (follicular) or hinder (luteal) heat loss mechanisms, and subsequently performance owing to their influence on core temperature [42].

This inter-system relationship is further complicated by women’s ever-changing endocrine profile, which alters naturally with maturation [43] and the use of hormonal contraceptives [44]. Thus, it is logical that while gaps in our understanding of this area remain, researchers should be encouraged to collect and report the menstrual cycle orientation of participants. Such transparency would beneficially expand the pool of scientific data relevant to women in specific life stages and enable the research community to maximize the work’s potential via data aggregation. Notably, the reporting of menstrual orientation did not occur in over 70% of the articles reviewed.

More evidence is needed to conclusively support or refute the effects of the menstrual phase on the rate of heat loss or onset threshold during exercise in hot environments [18, 19, 45]. For example, in recreationally active women, time to fatigue in hot, humid conditions [45], and exercise tolerance time in a hot, dry environment [46] were lower in the luteal than the follicular phase. More recent works have found that female reproductive hormones have trivial effects on the thermoregulatory system and exercise performance in the heat [18–20]. However, these articles, among others, acknowledge the need for continued investigation into both eumenorrheic women and hormonal contraceptive users [21, 38]. Martin and colleagues [47] showed that hormonal contraceptive use, especially oral contractive pill use, is highly prevalent in athletic women. While groups of oral contraceptive pill users were the second most frequently studied menstrual orientation identified in the literature reviewed in the current investigation, the proportion of these studies does not reflect the growing prevalence of oral contraceptive use nor the demand for research on exogenous, synthetic hormones [47].

A greater proportion of the articles that identified menstrual orientation also reported that the menstrual phase was controlled. This is a positive result as in controlling for phase, hormonal variability is reduced, and the effects of divergent hormonal concentrations can be examined at specific points in a cycle [48]. It should be noted that it is not always necessary to control the menstrual phase, for instance, when reproductive hormones (natural or synthetic) have been identified assuredly to have little or no effect on the study’s primary outcome measure. However, correctly controlling for the menstrual phase is essential to improve the internal validity of the research [49]. Inconsistent findings between studies could result from alternate phase verification methods or testing on different days or phases of the cycle [32, 50].

Recent methodological recommendations and guides to best practice aim to minimize this study heterogeneity and improve the quality of future research involving women [48, 49]. For example, combining calendar counting with urinary ovulation tests and serial blood sampling [51] and tracking participants’ menstrual cycle characteristics ≥2 months before testing improves the accuracy of phase identification [48]. Unfortunately, access to the necessary facilities, cost of analysis, and an extended data collection timeline may restrict adherence to this advice. In the case of hormonal contraceptive users, different forms, types, and steroid concentrations, along with individual exogenous-endogenous hormone interactions, must also be considered [48, 52]. The meticulous control measures and methodological considerations necessary to include women in thermoregulatory research and correctly control for menstrual phase may have deterred scientists from involving more women in their research over the past decade (Fig. 2, Table 1). However, without appropriate amendments to current research practice, aspects of female physiology—that meaningfully influence temperature regulation—will remain questioned or unidentified.

Based on the findings of this analysis, how can researchers best serve to increase women’s representation and scientific knowledge? Firstly, recognition that while representation can be improved, women deserve to be included in research purposefully where their outcomes can contribute to scientific knowledge, not merely for the sake of equal representation. Secondly, researchers should consider whether previous research conclusively or inconclusively identifies differences between the sexes in their primary outcome variable(s) during the research development and design stage. For topics where sex differences remain inconclusive, designing a women-only or study with men and women would best serve to increase the scientific knowledge on women. Further, if both sexes are examined, the data of men and women should not be aggregated as the results could identify whether a sex difference on the topic exists. Finally, studies including women must subsequently choose whether to control for menstrual orientation and phase. Again, researchers should consider if natural or synthetic reproductive hormones have or have not conclusively been identified to influence the primary outcome variable(s).

In the current review, participants were most frequently tested in hormonal phases that exhibit a depressed hormonal profile when the effects of estrogen and progesterone on other physiological systems are minimized. Hence, it is reasonable to suggest that in some of these studies, testing was conducted in specific menstrual phases to reduce the potential confounding effects of reproductive hormones on the measure(s) of interest. This practice does not serve to improve our knowledge on this topic where more evidence on the relationship between the reproductive and thermoregulatory systems is required. For a comprehensive inventory of study design considerations and guidelines for testing women with different hormonal profiles see Elliot-Sale and colleagues’ work [48].

It is clear from our analysis that future investigations involving women should include larger sample sizes—the median sample of studies including both sexes was extremely small (men, *n* = 10; women, *n* = 6). For context, a sample size of 6 per group has a mere 24% power—and 10 per group 40% power—to detect an effect size of 0.8 (often defined as a large effect), given α = 5%. Sample size justifications (e.g., power analyses) require greater consideration as do analytical approaches that allow the evaluation of apparently null results (e.g., equivalence testing, Bayes factors) [53, 54]. We appreciate that practical, logistical, and financial reasons may preclude the recruitment of larger samples. When small samples are unavoidable, we recommend that researchers think carefully about their smallest effect size of interest [54], where possible, use equivalence testing to help draw substantive conclusions from a study’s results [54]; and consider the use of fully Bayesian methods, to leverage the benefits of setting informative prior distributions [53], based on previous literature or expert elicitation [55]. Data aggregation and meta-analyses are additional strategies that can help alleviate small sample size concerns and improve sample bias corrections. Though methodological inconsistencies likely prevent most current studies from being pooled, this may be possible with future works [56]. The standardized reporting of individual hormonal concentrations may improve confidence when pooling results and assist with study interpretation (e.g., explaining outliers and unexpected results).

## Conclusion

This review highlights the underrepresentation of women in the exercise thermoregulation literature between 2010 and 2019. This underrepresentation belies the growth in women active in sport and industry, and also the pressing need to expand the scientific evidence relevant to women. Further, the infrequency of reporting menstrual orientation and menstrual phase in the articles reviewed has provided an insightful perspective on current research practices and where improvements can be made. To facilitate increased knowledge in women’s exercise thermoregulation research, the following practices could be improved: (i) transparent reporting of menstrual orientation, (ii) researchers should be conscious of the gaps in women’s research and consider the purposeful inclusion of women in their work, (iii) improvements in data transparency for subsequent aggregation, and finally (iv) appropriate study planning, design, and analysis is vital.

## Supplementary Information

**Additional file 1.**

**Additional file 2.**

**Additional file 3.**

## Data Availability

The data that reproduce the findings of the study can be accessed at https://github.com/katehutchins/female-thermoreg-review.

## Abbreviations

CI: Credible interval
IQR: Interquartile range
MCMC: Markov chain Monte Carlo
ZOIB: Zero- and one-inflated beta
