# Supplementary material for: Female (Under) Representation in Exercise Thermoregulation Research
Source: Sports Med Open. 2021 Jun 22;7:43. doi: 10.1186/s40798-021-00334-6 (PMC8219822; doi:10.1186/s40798-021-00334-6)
Supplement: Supplementary file 1 — Additional file 1. [file 40798_2021_334_MOESM1_ESM.pdf]

Supplementary 1. List of search terms, Boolean operators and limits utilised in search strategy

Searched with OR	**Exercise Terms**<br><br>(exercise ,“physical activity”, fitness, performance, running, run, sprint, sprinting, walking, walk, jog, jogging, swim, swimming, cycle, cycling, bicycling, bike, riding, "team sport", "individual sport", athletics, sport, sports, train, training, aerobic, anaerobic, gymnastics, gym, “resistance training”, lifting, climb, climbing, “ball sports”, activity, active, “social activity”, “social activities”, “recreational activity”, “recreational activities”, “physical exertion”, “stair climb”, “stair climbing”, swimming, “physical conditioning”, “stretching”, “plyometric”, “high intensity interval training”, “endurance training”, “circuit based”, “recreational sport”, “recreational sports”, “exercise therapy”, “physical fitness”, “physical endurance”, “cardiovascular fitness”, “racquet sports”, “water sports”, “snow sports”, wrestling, “weight lifting”, volleyball, “track and field”, soccer, skating, tennis, skiing, mountaineering, “martial arts”, “Tai Ji”, hockey, golf, football, boxing, basketball, rugby, cricket, baseball, badminton, yoga, pilates)
Searched with AND	
Searched with OR	**Thermoregulatory Terms**<br><br>("core temperature", "body temperature", "skin temperature", "rectal temperature", "esophageal temperature", "oesophageal temperature", "intestinal temperature", "tympanic temperature", "tissue temperature", "internal temperature", "muscle temperature", “blood temperature”)

**Limits**	
Search Field:	Title and Abstract
Publication Date:	2010/01/01-2019/12/31
Language:	English
Availability:	Abstract Available
