# Supplementary material for: Female (Under) Representation in Exercise Thermoregulation Research
Source: Sports Med Open. 2021 Jun 22;7:43. doi: 10.1186/s40798-021-00334-6 (PMC8219822; doi:10.1186/s40798-021-00334-6)
Supplement: Supplementary file 2 — Additional file 2. [file 40798_2021_334_MOESM2_ESM.pdf]

Title: Female (Under) Representation in Exercise Thermoregulation Research

Journal: Sports Medicine Open

Authors

Kate P Hutchins[1], David N Borg[2], Aaron JE Bach[3], Joshua J Bon[4,5], Geoffrey M Minett[1], Ian B Stewart[1]

Affiliations

[1]Queensland University of Technology (QUT), School of Exercise and Nutrition Sciences, Institute of Health and Biomedical Innovation, Brisbane, Australia.

[2]Griffith University, Menzies Health Institute Queensland, The Hopkins Centre, Brisbane, Australia.

[3]Griffith University, The National Climate Change Adaption Research Facility (NCCARF), Gold Coast, Australia.

[4]Queensland University of Technology (QUT), School of Mathematical Sciences, Brisbane, Australia.

[5]Australian Centre of Excellence for Mathematical and Statistical Frontiers (ACEMS), Brisbane, Australia.

Corresponding Author

Kate P Hutchins

Email: k.hutchins@qut.edu.au

Supplementary 2. Mean (95% credible interval) proportion of female participants included in exercise thermoregulation research between 2010 and 2019.

Year	Mean	95% lower	95% upper
2010	11.55%	9.17%	14.29%
2011	12.14%	9.98%	14.54%
2012	12.76%	10.86%	14.84%
2013	13.40%	11.75%	15.20%
2014	14.07%	12.58%	15.67%
2015	14.77%	13.35%	16.24%
2016	15.50%	13.97%	17.06%
2017	16.25%	14.48%	18.09%
2018	17.02%	14.87%	19.26%
2019	17.82%	15.22%	20.55%
