# Supplementary material for: Female (Under) Representation in Exercise Thermoregulation Research
Source: Sports Med Open. 2021 Jun 22;7:43. doi: 10.1186/s40798-021-00334-6 (PMC8219822; doi:10.1186/s40798-021-00334-6)
Supplement: Supplementary file 3 — Additional file 3. [file 40798_2021_334_MOESM3_ESM.pdf]

Title: Female (Under) Representation in Exercise Thermoregulation Research

Journal: Sports Medicine Open

Authors

Kate P Hutchins[1], David N Borg[2], Aaron JE Bach[3], Joshua J Bon[4,5], Geoffrey M Minett[1], Ian B Stewart[1]

Affiliations

[1]Queensland University of Technology (QUT), School of Exercise and Nutrition Sciences, Institute of Health and Biomedical Innovation, Brisbane, Australia.

[2]Griffith University, Menzies Health Institute Queensland, The Hopkins Centre, Brisbane, Australia.

[3]Griffith University, The National Climate Change Adaption Research Facility (NCCARF), Gold Coast, Australia.

[4]Queensland University of Technology (QUT), School of Mathematical Sciences, Brisbane, Australia.

[5]Australian Centre of Excellence for Mathematical and Statistical Frontiers (ACEMS), Brisbane, Australia.

Corresponding Author

Kate P Hutchins

Email: k.hutchins@qut.edu.au

Supplementary 3. Mean (95% credible interval) parameter estimates from the zero- and one-inflated beta distribution model.

Model component	Parameter	Notation	Mean	95% lower	95% upper
logit(E(y|$y \in (0,1)$))	Intercept	$\alpha_{(0,1)}$	-0.53	-0.71	-0.36
	Year	$\beta_{(0,1)}$	0.01	-0.02	0.04
logit(Pr(y = 0))	Intercept	$\alpha_{\{0\}}$	1.17	0.93	1.42
	Year	$\beta_{\{0\}}$	-0.07	-0.11	-0.03
logit(Pr(y = 1))	Intercept	$\alpha_{\{1\}}$	-1.50	-2.04	-0.99
	Year	$\beta_{\{1\}}$	-0.01	-0.10	0.07
Shape	Shape	d	1.77	1.63	1.90

Note. Regression coefficients are reported on the logit scale.
Pr = Probability
d = Regression coefficient in the linear predictor for the sum of the two shape parameters in the beta distribution.
